# Amniotic band syndrom at Bobo Dioulasso university teaching hospital (Burkina-Faso): about two cases

**DOI:** 10.11604/pamj.2015.22.187.7863

**Published:** 2015-10-23

**Authors:** Cyprien Zaré, Ibrahim Alain Traoré, Patrick Wendpuoiré Hamed Dakouré, Salif Gandéma, Bakary Gustave Sano, Lazard Bouma Bénao, Hermann Belemlilga, Nassirou Yabré

**Affiliations:** 1 Departement of General Surgery University Hospital Teaching Souro Sanou (CHUSS), Bobo-Dioulasso, Burkina Faso; 2 Department of Anesthesiology University Hospital Teaching Souro Sanou (CHUSS), Bobo-Dioulasso, Burkina Faso; 3 Service Orthopaedic and Traumatology University Hospital Teaching Souro Sanou(CHUSS), Bobo-Dioulasso, Burkina Faso; 4 Service of Functional Rehabilitation University Hospital Souro Sanou (CHUSS), Bobo-Dioulasso, Burkina Faso

**Keywords:** Amniotic band syndrom, surgery, birth defects

## Abstract

Amniotic band syndrome is a rare congenital disorder. The authors report the first cases documented at Souro Sanou University Hospital in Bobo-Dioulasso (CHUSS) in 2 male new borns. The malformations found at birth, were worn only on limbs and were in the form of skin furrow necking with a major lymphedema downstream. In both cases, the constriction furrow at member pelvic was associated with a club foot and a pseudosyndactyly in one case. Surgical treatment consisted of a section of the constrictor ring and a Z-plasty. The functional outcome was satisfactory with the acquisition of a plantar support for both children. Through these two observations, epidemiological, diagnostic, and particularities of the management of this condition are discussed in the Burkina-Faso.

## Introduction

Amniotic band syndrome (ABS) which is a clinical entity is a set of various deformities ranging from minor cutaneous sulcus to major (visceral, craniofacial) [1, 2]. It is a rare disease [3, 4]. Antenatal diagnosis is difficult in our communities and the diagnosis is done only at birth. There are several therapeutic options in either prenatal or postnatal cases. The African literature concerning the subject is poor. Through two cases in Bobo-Dioulasso University Teaching Hospital, we describe the epidemiological and diagnostic aspects of ABS; and discuss the management of this disease in Burkina Faso.

## Patient and observation

### Observation N^o^ 1

**Figure 1:**
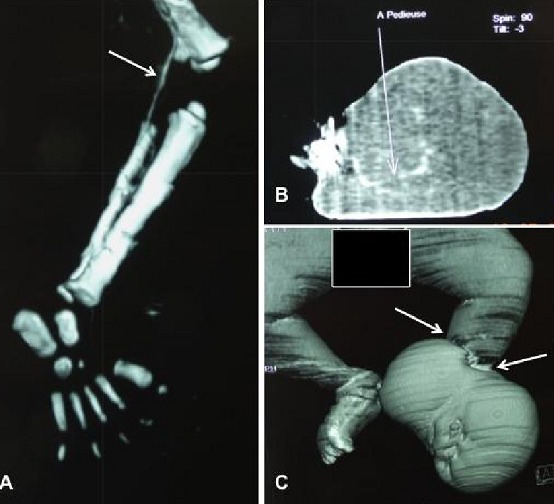
CT angiography of pelvic members in an infant of 04 months displaying amniotic bands left. A) note the left popliteal artery correctly injected, bone structures without fault; B) the vascularization is normal with a note highlighting the left pedal artery; C) necking with downstream lymphedema

**Figure 2:**
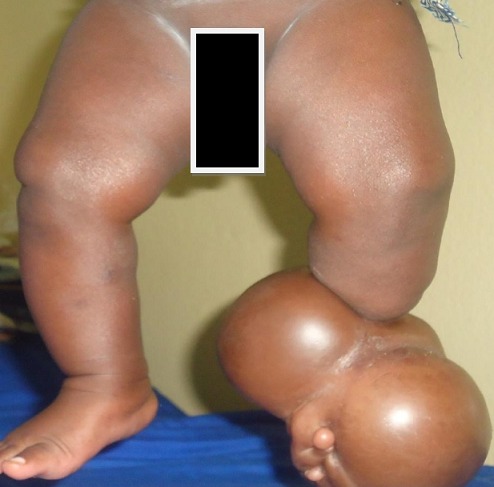
Children of 4months of life. Cutaneous circulatory constrictorring associated with a large lymphedema and left club foot downstream of the constriction. No skin lesions are objectified

**Figure 3:**
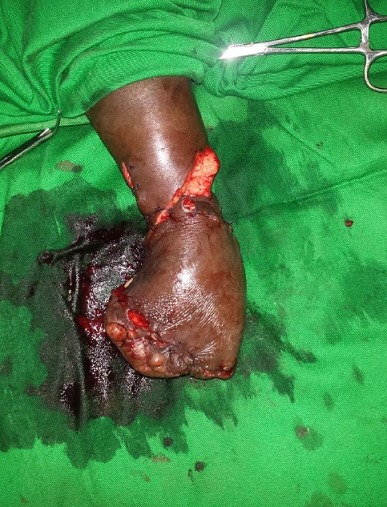
Per-Intervention aspect of the foot after the necking section and Z-plasty

**Figure 4:**
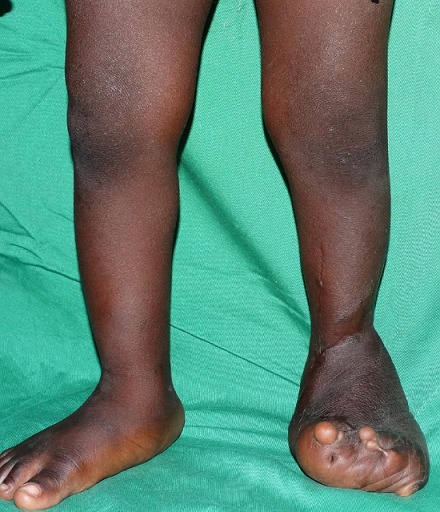
Appearance of the malformed foot with a plantar support two postoperative years

A four-month-old baby boy, from a monochorionic-monoamniotic pregnancy, born after a full-term and normal delivery was admitted in general surgery consultation for congenital left foot deformity. He is the seventh in a family of seven children all alive and apparently healthy. No ultrasound was done during the pregnancy. There was no notification of family congenital pathology. During the examination we noticed on the lower third of the left leg a malformation characterized by a circulatory constrictor ring of foot responsible for a major lymphedema in downstream without signs of cutaneous suffering. This deformity was associated with ipsilateral clubfoot. The left foot plain radiography requested showed no bone malformation. Abdominal ultrasound looking for associated visceral malformations was normal. The foot's Computed Tomography Angiography has not recovered vascular anomaly including the dorsalis pedis artery (Figure 1). The diagnosis of ABS isolated without severe sign was retained (Figure 2). After a satisfactory weekly follow-up, the child underwent surgery at the age of six months. The intervention consisted under general anesthesia with orotracheal intubation and withers to the root of the pelvic limb, in a section of the constrictor ring and a Z-plasty skin (Figure 3). Functional rehabilitation of the member began a month after the surgery. It consisted of an activity of articular motions recovery of a working of recovery of range's motion, muscular revival and rehabilitation to walking. At two years of surgery, walking was possible with a full plantar support, offering a functional capacity close to normal (Figure 4).

### Observation N^o^ 2

**Figure 5:**
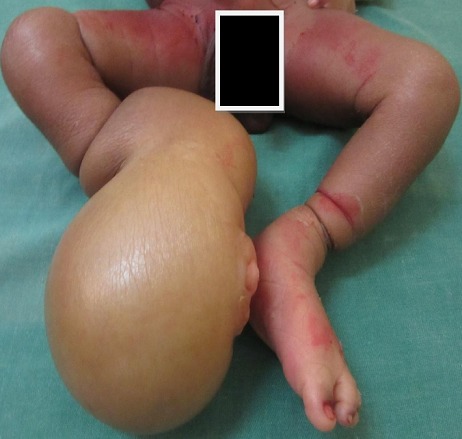
Aspects of pelvic members with multiple grooves constrictions including the right leading to a large lymphedema

This is a one-day-old baby boy, from a monochorionic monoamniotic pregnancy, born after a full-term and normal delivery, and then transferred to from the pediatric ward for multiple congenital malformations in the limbs. In medicals histories it was noted that it is the first child. During the pregnancy care no obstetric ultrasound had been performed. Clinical examination revealed multiple skin furrows located on both legs and a syndactyly of the second and third left fingers. The furrows were bilateral in each leg and both located in the right leg have led to a significant distal lymphedema with no signs of skin necrosis and were associated with ipsilateral club foot (Figure 5). The search of associated visceral bone and vascular malformations by abdominal ultrasound, plain radiographs, and CT angiography of members was negative. The child was operated at six months of life and received a surgical procedure that involved a section of the furrows and skin Z-plasty. The postoperative course was uneventful. Physical therapy was performed one month after surgery to obtain an acceptable function of the right pelvic member.

## Discussion

ABS is a rare disease in our practice (2 documented cases). This scarcity was noted by Sentilhes and al. and Fries and al. who reported an incidence between 1/1200 to 1/15000 [3, 4]. In our series, it was about two male new born. In the literature, no gender predominance was noted [5, 6]. The lack of obstetric ultrasound in our working conditions or often not within the financial reach of women during follow-up of pregnancy, practiced by some authors could facilitate antenatal diagnostic [3, 7, 8]. All of our 2 cases were diagnosed at birth on the recognition of a malformation syndrome. He basically acted limb deformities characterized by skin necking furrows. Which literature, this observation was also made by some authors [9, 10]. Other lesions may be associated, including club foot, convex feet, and bone defects [1]. This is the case of our observations where a clubfoot was associated in 02 cases and in 01 cases syndactyly. In the literature, emergency surgery within 48 hours of life advocated by Sentilhes L et al [3], can't be done in our working conditions. Indeed our two children were operated at six months of life, as precaution of good anesthetic practice on newborn in our midst. This precaution is justified by the lack of equipment, suitable materials and especially the lack of training of health agents workers that make anesthesia in children in general, and the newborn in particular, dangerous and risky in our context works environment. The importance of lymphedema related to necking was the main indication for surgery in postnatal birth in our two cases. The absence of severity's signs (ischemia, skin necrosis, bone defect). During diagnosis and follow-up allowed us to delay surgery for good practice of anesthesia. As recommended by most of the authors [1–3], we practiced a section of the ring and the groove plasty via multiple Z-incisions. As for some authors, surgery has allowed us to obtain a functional capacity of members close to normal [2].

## Conclusion

The ABS is uncommon in our community. The lack missing of obstetrical ultrasound as a systematic means of monitoring pregnancies in our practice does not permit antenatal diagnosis. However the clinical diagnosis is easy at birth. Surgical treatment is done at six months of life in order to minimize anesthetic risks in developing countries like ours. The functional result was satisfactory for both children, they acquire a plantar support. An improving of our anesthesia practice conditions will permit an early management of this disease.
